# HIV-1 infection recency among individuals newly diagnosed with HIV in Greater Gaborone, Botswana

**DOI:** 10.1097/QAD.0000000000004239

**Published:** 2025-07-25

**Authors:** Natasha O. Moraka, Charity Ralegoreng, Goitseone M. Lemogang, Richard Makwakwa, Marea N. Pema, Patrick T. Mokgethi, Ontlametse T. Choga, Irene Gobe, Margaret Mokomane, Dorcas Maruapula, Salang T. Moutswi, Laone Rabatoko, Queen Leteemane, Vanessa Strachan-Amaro, Phenyo Sabone, Terence Mohammed, Sikhulile Moyo, Simani Gaseitsiwe

**Affiliations:** a Botswana Harvard Health Partnership; b Faculty of Health Sciences, School of Allied Health Professions, University of Botswana; c Department of Biological Sciences, University of Botswana, Gaborone, Botswana; d Department of Immunology and Infectious Diseases, Harvard University T.H Chan School of Public Health, Boston, MA, USA; e School of Health Systems and Public Health, Faculty of Sciences, University of Pretoria, Pretoria; f Division of Medical Virology, Department of Pathology, Faculty of Medicine and Health Sciences. Stellenbosch University, South Africa.

**Keywords:** Botswana, Gaborone, HIV type-1, new HIV-1 diagnoses, recent infection

## Abstract

**Objective:**

We characterized individuals with HIV type 1 (HIV-1) recent infection using a two-step recent infection testing algorithm (RITA) in recently diagnosed, antiretroviral therapy (ART)-naive individuals within the Greater Gaborone area in Botswana.

**Design:**

Plasma samples from a prospective longitudinal cohort study of individuals recently diagnosed with HIV within the Greater Gaborone area (2023–2024), the Tekodiso study, were used.

**Methods:**

Recent infection classification was determined using Limiting Antigen Avidity (LAg-avidity), as well as HIV viral load greater than 1000 copies/ml. LAg-normalized optical density (ODn) 1.5 or less represented a recency window of within 130 days postinfection. HIV viral load in plasma was quantified by Abbott m2000sp/Abbott m2000rt.

**Results:**

A total of 157 participants were included in this analysis. Median age at enrolment was 34 years (Q1, Q3: 27, 41), and majority 102 (65%) were female. The median log_10_ HIV viral load was 4.6 copies/ml (Q1, Q3: 3.9, 5.2). A total of 12 of 157: 7.6% [95% confidence interval (CI) 4.0–13] individuals were classified as having a recent infection. Recent infection was not associated with age, employment status, or nationality. We observed a lower likelihood of recent HIV infection with secondary or higher education level (OR = 0.1; 95% CI: 0.1– 0.9).

**Conclusion:**

We report a 7.6% rate of HIV recent infection by LAg-based RITA in recently diagnosed ART-naive individuals in Botswana. Our results highlight the need for continued HIV infection surveillance to improve targeted interventions for the prevention of HIV acquisition within the country.

## Introduction

HIV incidence continues to decline worldwide; however, there is 1.3 million new infections of adults and children annually [1,2]. The aspirations of ending HIV as a public health threat by 2030 continue to be off target with the current rates and trajectory of new infections [1]. Characterizing new infections is, therefore, important for establishing and monitoring prevention strategies for groups at risk of HIV acquisition within countries. The ‘gold’ standard method for the assessment of HIV incidence is setting up longitudinal cohort studies [3]. Unfortunately, this methodology is extremely costly and time-consuming; not feasible for low-income to middle-income countries, highlighting the need for more cost-effective and generalizable measures of incidence within countries.

Several measures of cross-sectional incidence have been evaluated worldwide. The WHO currently recommends that countries with HIV prevalence greater than 5% and incidence of greater than 0.3% include the use of biomarkers to test for incidence within population-based surveys [4]. Furthermore, WHO recommends coupling biomarker assays with HIV viral load results to reduce misclassification of individuals as recent infections [4]. A previous study carried out HIV recency algorithm testing that included the limiting antigen (LAg) assay and HIV-1 VL data [5]. This recent infection testing algorithm (RITA) produced a low false recency rate (FRR) in the Botswana population sampled in the mid-2000s [5], making it suitable for us to now evaluate cross-sectional recency of infection within the ‘Treat All’ era in the Botswana population.

Botswana has a robust HIV treatment program, which has been at the forefront of reducing new HIV diagnoses, as well as achieving 98% viral suppression among its population of people with HIV (PWH) who know their status and are on ART [6]. Despite this commendable success, the number of incident HIV cases in Botswana is still high, with an estimated 4300 new HIV infections occurring annually [7]. Moreover, the number of PWH who are identified as recent infections has not been established since the implementation of ‘Treat All’.

We characterized individuals identified as having HIV-1 recent infection using a two-step RITA in recently diagnosed, antiretroviral therapy (ART)-naive individuals enrolled from government clinics within the Greater Gaborone area in Botswana, and evaluated factors associated with HIV recent infection.

## Methods

### Study population and sampling

Whole blood samples were collected during an ongoing longitudinal cohort study, the ‘Tekodiso study’. Samples from individuals who were recently HIV diagnosed within government clinics in the Greater Gaborone area in Botswana (October 2023–November 2024) were collected. All samples were collected using a consecutive census approach, where all participants diagnosed with HIV by double rapid HIV testing were included in the study on the same day of diagnosis before ART initiation.

### Ethical considerations

The ‘Tekodiso’ study has been approved by the Botswana Ministry of Health's Research and Development Committee (HRDC), the University of Botswana Institutional Review Board (IRB) as well as the Greater Gaborone District Health Management Team (GGDHMT). All participants were taken through a written consenting process, where eligibility was defined as being 18 years or older and testing HIV positive for the first time.

### HIV infection recency

Recent infection classification was determined using Limiting Antigen Avidity (LAg-Avidity) assay (Sedia HIV-1 LAg-Avidity EIA; Sedia Biosciences Corporation, Portland, Oregon, USA) [8] as per the manufacturer's instructions. We used a cutoff of 1.5 normalized optical density units (ODn) to define avidity, the ODn 1.5 or less represented the recency window of 130 days postinfection.

### HIV-1 viral load testing

HIV-1 viral load in plasma was quantified using Abbott m2000sp/Abbott m2000rt (Abbott Laboratories, Wiesbaden, Germany). The lower limit of detection for this assay is less than 40 copies/ml.

### Viral load-based Lag recent infection testing algorithm

We used a two-step RITA to classify participants into recent and long-term HIV-1 infections. LAg ODn 1.5 or less represented Lag ‘recency’, while LAg ODn greater than 1.5 represented LAg ‘long term’ infection. Further classification was combined with HIV-1 RNA/VL results; where viral load greater than 1000 copies/ml was defined as virologic failure and when coupled with Lag ODn 1.5 or less represented ‘recent’ infection in the viral load-based LAg RITA. viral load less than 1000 copies/ml coupled with LAg ODn greater than 1.5 were defined as ‘long-term’ infection in the viral load-based LAg RITA.

### Statistical analysis

Descriptive statistics were utilized to describe the frequency of individual variables for baseline participant characteristics. Results are presented as medians with interquartile ranges (Q1, Q3) for continuous variables and absolute numbers with percentages for categorical variables. Univariate logistic regression analysis was further performed to test for association of individual variables with HIV infection recency status. Selected variables were age, gender, HIV RNA load, baseline CD4^+^ T cell, education level (primary/no education vs secondary education and above) and non-Motswana nationality; these variables were selected *a priori* and results are presented as odds ratios with their 95% confidence intervals.

## Results

### Baseline characteristics

A total of 157 Tekodiso study participants were included in this analysis. Median age at enrollment was 34 years (Q1, Q3: 27, 41) (Table 1). Most study participants were female (65%) and single (88.5%). The median log_10_ HIV viral load was 4.6 copies/ml (Q1, Q3: 3.9, 5.2), 12 (7.6%) participants had undetectable HIV viral load at baseline. CD4^+^ T-cell count data was extracted from the Botswana National ART data warehouse and was available for only 112 participants; median CD4^+^ cell count was 334 cells/μl (Q1, Q3: 204, 501). The majority of participants had gone through secondary education (72.6%) and were employed (69.4%) (Table 1).

**Table 1:** Baseline characteristics of Tekodiso study participants.

Baseline characteristics (*N* = 157)	
Age (years) [median (IQR)]	34 (27, 41)
Gender [*n* (%)]	
Female	102 (65.0)
Male	55 (35.0)
HIV RNA load (log_10_ copies/ml) [median (IQR)]	4.6 (3.9, 5.2)
Baseline CD4^+^ cell count (cells/ml) [median (IQR)]	334 (204,501)
Marital status [*n* (%)]	
Married	18 (11.5)
Single	139 (88.5)
Highest education level [*n* (%)]	
None	1 (0.6)
Primary/nonformal	11 (7.0)
Secondary	114 (72.6)
Tertiary	31 (19.8)
Recruitment site [*n* (%)]	
Bontleng Clinic	38 (24.2)
Lesirane Clinic	51 (32.5)
Mogoditshane Clinic	14 (8.9)
Nkoyaphiri Clinic	35 (22.2)
Tlokwenng Main Clinic	17 (10.8)
Mafitlhakgosi Clinic	2 (1.3)
Non-Motswana nationality [*n* (%)]	42 (26.8)
Employed [*n* (%)]	
No	48 (30.6)
Yes	109 (69.4)

CD4, Cluster of Differentiation 4; IQR, interquartile range; RNA, ribonucleic acid.

### Recent infection by viral load-based recent infection testing algorithm

**Fig. 1:**
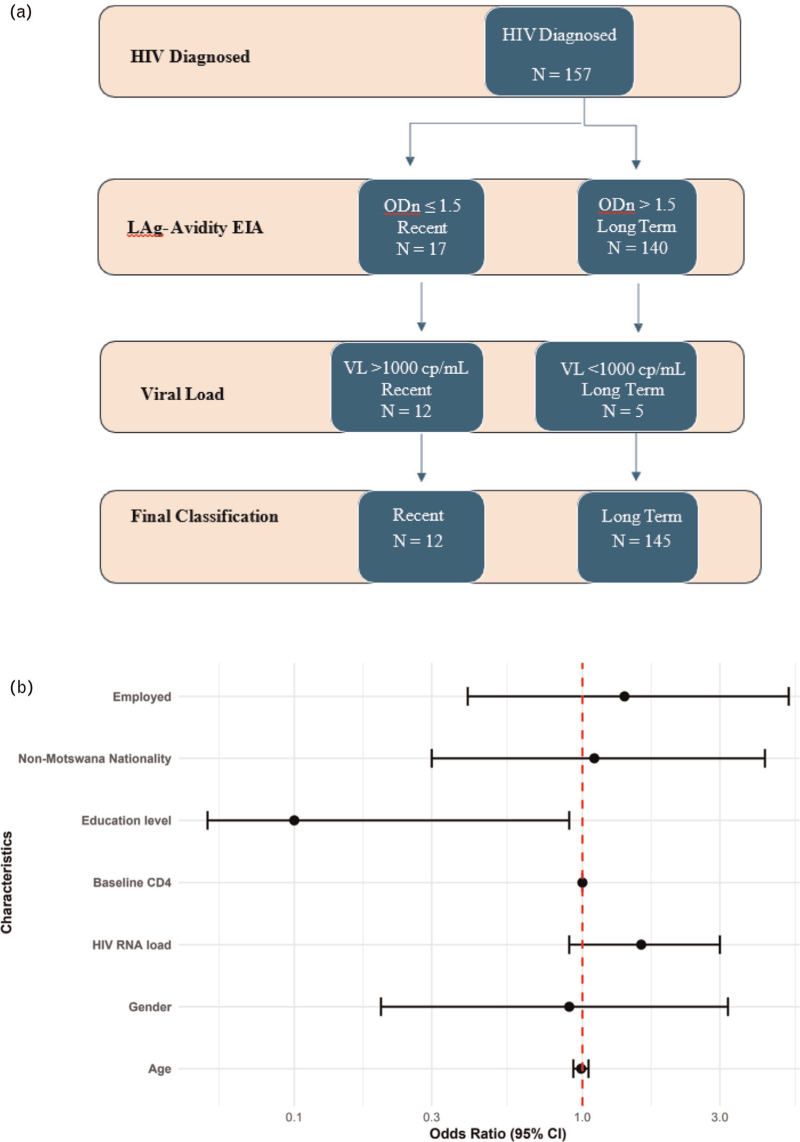
HIV recent infection testing algorithm using LAg and HIV-1 viral load testing to determine recent infection and participant demographics associated with HIV infection recency in newly HIV- diagnosed individuals in the Greater Gaborone area, Botswana.

A total of 157 participants were screened for HIV recency using Limiting Antigen Avidity (LAg-Avidity) assay (Sedia HIV-1 LAg-Avidity EIA; Sedia Biosciences Corporation), 17 (10.8%) of the participants were preliminarily classified as recent infections with ODn 1.5 or less (Fig. 1a). Of these, five participants had HIV viral load of less than 1000 copies/ml and were, therefore, re-classified as long-term infections (Fig. 1a). Thus, using the two-step VL RITA, 7.6% (95% CI 4.0–13.0) of individuals were classified as recent infections (Fig. 1a). Among these, majority (75%) are Batswana and 67% are female.

(a) Classification of HIV infection recency using ODn from LAgEIA and HIV RNA load of study participants at the time of HIV diagnosis. (b) A univariate logistic regression analysis of associations between recent HIV infection and participant baseline characteristics in the Tekodiso study.

### Characteristics of recent infections

Having a higher HIV RNA load was associated with an increased likelihood of recent infection [odds ratio (OR) = 1.6; 95% CI: 0.9– 3.0], although this was not statistically significant (Fig. 1b). We do not observe any correlation of recent infection with participant's sex, age, employment status, or nationality. We observe a likelihood that having received secondary or higher education reduced the risk of recent HIV infection (OR: 0.1, 95% CI: 0.1–0.9), *P* = 0.03 (Fig. 1b). Upon adjusting for potential confounding variables (age, gender and baseline HIV-RNA), having received secondary education still showed to reduce risk of HIV infection recency (OR: 0.1, 95% CI: 0.02–0.9), *P* = 0.03 (data not shown).

## Discussion

The rates of HIV infection recency in the sub-Saharan African region have been conducted in very few studies since the introduction of same-day ART initiation and preexposure prophylaxis. This article provides a cross-sectional insight into the effectiveness of the ‘Treat-All’ strategy in Botswana by providing the first dataset to evaluate recent HIV-1 infection among people diagnosed with HIV within the greater Gaborone area between October 2023 and November 2024. Our data presents information from a unique cohort of individuals with same-day diagnosis of HIV-1 from six local government clinics.

Using an HIV RNA load-based RITA, we report a 7.6% infection recency rate representing a recency window of within 130 days postinfection among study participants. Our results are concordant with a recent house-to-house campaign study in Lesotho, which reported an HIV recent infection rate of 7% among seroconverters within the VIBRA cohort [9]. Similarly, a study in Kenya evaluated HIV recency using a Lag-based RITA on stored samples from people living with HIV from their 2007 Kenya AIDS Indicator Survey [10], they reported a 6.2% HIV infection recency rate out of 1025 study participants. In Ethiopia, a retrospective analysis conducted on an HIV case-based surveillance dataset between July 2019 and June 2022 reported a recent HIV infection rate of 11.3% from 786 participants [11]. Overall, our results are comparable with what is observed in the region and lower compared to what is observed in other countries outside sub-Saharan Africa [12,13]. The main differences observed in studies evaluating recent infection can be attributed to differences observed in HIV incidence rates across populations, discrepancies in RITA, as well as inequalities in the number of recent HIV infections diagnosed among newly diagnosed HIV cases [11]. These differences highlight the need for validation of accurate and effective RITAs to use to identify trends in epidemiology especially in nonsurveillance settings [14].

We do not observe many associations between baseline participant characteristics and recent HIV infection. These results are in concordance with what has been found in the region [9,10]. A nationally representative household survey conducted in Uganda in the early 2000s showed no significant difference between being recently HIV-infected and age, urban residence, socio-economic status, age at sexual debut, pregnancy, religion, and alcohol intake or employment [15]. In contrast, a more recent study in Ethiopia reported that younger age of 15–24 years was associated with seven times the risk of HIV recent infection [11]. Furthermore, it is important to note that when looking at results from the recent Botswana AIDS Impact Survey (BIASV) [6], younger people in the age bracket of 15–24 years form most of the population who do not know their status within the population. It is, therefore, possible that these individuals could also have a significant role in HIV transmission/new infections; this has led to several efforts and campaigns to get young members of the community to test for HIV by establishment of youth-friendly services in local public health facilities.

Generally, due to late presentation of males to healthcare facilities, HIV diagnoses are usually detected more among females than males. We report a significantly higher proportion of females diagnosed with HIV within the Tekodiso cohort although these were not associated with HIV recent infection, these results are in contrast with what has been observed in the region [10,15].

Strong associations were observed between having a lowered risk of HIV recent infection and having received secondary and tertiary education. The positive impact of receiving education on HIV incidence has been reported in several studies [16–18]; however, some studies like the one carried out in Ethiopia show that the odds of recent HIV infection are double fold among individuals who receive secondary education and above compared to clients with primary or no formal education [16].

Our study had several limitations, the lack of a denominator of how many people were tested in the facilities where 157 of them were HIV diagnosed limited our ability to estimate HIV incidence and calculate the rates of recent diagnosis in the region. Although this may be seen as a strength in the analysis, the Tekodiso study is being conducted in the capital city of Botswana, which may have an impact on generalizability of results outside urban areas in the country. We also did not screen for other risk factors of HIV recent infection including number of sexual partners, use of contraception (including condom use and safe male circumcision) or prevalence of other sexually transmitted infections. Furthermore, the inability to confirm antiretroviral drug exposure limits confirmation of participants as true recent diagnoses. Although these limitations may hinder accurate generalizability of our results, this analysis provides insights on noncontrolled government facility settings, which may be more representative of what epidemiologic trends occur within the greater Gaborone area.

We report for the first time since implementation of same-day HIV diagnosis and treatment in Botswana, a 7.6% rate of HIV recent infection amongst individuals recently diagnosed with HIV within the greater Gaborone area. Our results confirm that there is ongoing transmission of HIV despite all interventions put in place within the country. These results highlight the need to continue with HIV infection surveillance to improve on targeted interventions for prevention of HIV acquisition.

## Acknowledgements

We would like to thank the Tekodiso study participants as well as our study recruitment sites within the greater Gaborone DHMT. We would also like to acknowledge the Botswana Harvard Health Partnership regulatory department: Mr. N Seonyatseng, Mrs. A. Bafana, and Mrs T. Sekoto who have been instrumental in study proceedings.

Authors’ contributions: N.O.M.: concept design, Tekodiso study conception and coordination, experimental tests, data analysis and writing of the manuscript with all supporting documents.

C.R.: laboratory recency screening. G.M.L.: laboratory recency screening. R.M.: laboratory HIV RNA testing. M.N.P.: laboratory recency screening. P.T.M.: manuscript and concept review.

O.T.C.: manuscript and concept review. I.G.: academic support for NOM and review of concept. M.M.: academic support for NOM and review of concept. D.M.: manuscript and concept review.

S.T.M.: Tekodiso study participant consent and recruitment. L.R.: Tekodiso study participant consent and recruitment. Q.L.: Tekodiso study participant consent and recruitment. V.A.: Tekodiso study participant consent and recruitment. P.S.: Tekodiso study data management.

T.M.: Tekodiso study laboratory management. S.M.: concept design and overall manuscript review. S.G.: concept design and overall manuscript review.

Sources of funding: This research was supported by the Sub-Saharan African Network for TB/HIV Research Excellence (SANTHE) which is funded by the Science for Africa Foundation to the Developing Excellence in Leadership, Training and Science in Africa (DELTAS Africa) programme [Del-22-007] with support from Wellcome Trust and the UK Foreign, Commonwealth & Development Office and is part of the EDCPT2 programme supported by the European Union; the Bill & Melinda Gates Foundation [INV-033558]; and Gilead Sciences Inc., [19275]. N.O.M, O.T.C and S.G were supported by the Fogarty International Center at the US National Institutes of Health (D43TW009610). N.O.M., S.G., and S.M. were partly supported through the Sub-Saharan African Network for TB/HIV Research Excellence (SANTHE 2.0) from the Bill and Melinda Gates Foundation (INV-033558). S.M. was supported by the Fogarty International Center at the US National Institutes of Health (K43TW012350). S.M. and O.T.C. were supported by the Trials of Excellence in Southern Africa (TESA III), which is part of the EDCTP2 program supported by the European Union (CSA2020-NoE-3104 TESAIII CSA2020NoE). The views expressed in this publication are those of the authors and not necessarily those of the funding agencies. The funders had no role in the study design, data collection, decision to publish, or preparation of this manuscript.

### Conflicts of interest

There are no conflicts of interest.
